# Sesame Oil Ameliorates Alanine Aminotransferase, Aspartate Aminotransferase, and Fatty Liver Grade in Women with Nonalcoholic Fatty Liver Disease Undergoing Low-Calorie Diet: A Randomized Double-Blind Controlled Trial

**DOI:** 10.1155/2022/4982080

**Published:** 2022-01-31

**Authors:** Masoumeh Atefi, Mohammad Hassan Entezari, Hamid Vahedi, Akbar Hassanzadeh

**Affiliations:** ^1^Department of Clinical Nutrition, School of Nutrition and Food Science, Isfahan University of Medical Sciences, Isfahan, Iran; ^2^Food Security Research Centre and Department of Clinical Nutrition, School of Nutrition and Food Science, Isfahan University of Medical Sciences, Isfahan, Iran; ^3^Department of Gastroenterology, School of Medicine, Shahroud University of Medical Sciences, Shahroud, Iran; ^4^Department of Epidemiology and Biostatistics, School of Health, Isfahan University of Medical Sciences, Isfahan, Iran

## Abstract

**Background:**

The type and amount of dietary fats play an important role in fat accumulation in the liver. Sesame oil (SO) is a good source of monounsaturated acids (MUFAs) and polyunsaturated fatty acids (PUFAs).

**Objective:**

This trial aimed at examining the effect of SO consumption on the levels of liver enzymes and the severity of fatty liver in women with nonalcoholic fatty liver disease (NAFLD) undergoing a weight loss diet.

**Methods:**

This randomized, double-blind, controlled trial was carried out on 60 women with NAFLD. Subjects were randomly assigned to the SO group (*n* = 30) and sunflower oil (SFO) group (*n* = 30), each person consuming 30 grams of oil per day for 12 weeks. All the participants received a hypocaloric diet (−500 kcal/day) during the study. Fatty liver grade and liver enzymes were assessed at pre- and postintervention phases.

**Results:**

53 patients completed the study. Significant reductions in body weight, body mass index (BMI), waist circumference (WC), and fatty liver grade were observed in both groups (*P* < 0.05). Following SO, significant decreases in serum aspartate and alanine aminotransferases (AST and ALT) were observed. After adjusting for confounders, ALT, AST, and fatty liver grade of the SO group were significantly reduced compared to the SFO group (*P* < 0.05). However, the changes in serum alkaline phosphatase (ALP) were not significant (*P* > 0.05).

**Conclusions:**

The desired effects of weight loss were reinforced by the consumption of SO through improving fatty liver severity and serum ALT and AST levels in NAFLD patients. Moreover, low-calorie diets may lead to favorable outcomes for NAFLD patients through mitigation of obesity and fatty liver grade.

## 1. Introduction

NAFLD is one of the most common liver diseases that are characterized by the accumulation of fat more than 5% of hepatocytes in the liver in the absence of alcohol consumption. If NAFLD is left untreated, it may lead to liver damages such as cirrhosis and hepatocellular carcinoma [1, 2]. NAFLD is known as the leading cause of death in patients associated with liver disease [3]. NAFLD is affecting many extrahepatic organs, including kidney, heart, and vessels, leading to notable morbidity and mortality [4]. Studies indicated that ALT predicts cardiovascular diseases (CVD), and individuals with NAFLD had a higher risk of heart disease than those without NAFLD [5, 6].

The prevalence of NAFLD is about 25% in the world and 33.9% in Iran [7, 8]. Diagnosis of NAFLD requires evidence of hepatic steatosis. Ultrasonography has been accepted as the first and best diagnostic method in investigations [9]. Also, the increase in the levels of liver enzymes including ALT, AST, and ALP is the key element in the diagnosis of NAFLD [10].

To date, pharmacological treatments have not been effective in controlling and improving NAFLD. The best treatment and strategy for these patients is lifestyle modification (having physical activity and a healthy diet) that can prevent the occurrence and progression of NAFLD [3, 11, 12]. The modification in the type and amount of dietary fat can affect lipid metabolism and fat accumulation in the liver [13, 14]. High-fat diet is well known as a major factor in the development of hepatic steatosis [15]. In contrast, the normal range of fat consumption containing MUFAs and PUFAs can have beneficial effects on the NAFLD [16].

SO is widely used as one of the health-promoting natural foods in Asian countries [17, 18], which is extracted from the seeds of *Sesamum indicum* L., belongs to the Pedaliaceae family [19], and is a good source of MUFAs (40%) and PUFAs (43%). Moreover, it contains vitamins B6, B12, and E, phenolic compounds such as phenols and flavonoids, lignans such as sesamin, sesamolin, and sesamol, and minerals such as calcium, iron, magnesium, copper, and phosphorus, which have several health-promoting effects leading to several physiological responses [17, 18, 20]. The physiological functions include the following: (1) increasing the antioxidant content and bioavailability of gamma-tocopherol; (2) lowering blood lipids and arachidonic acid; and (3) having hypocholesterolemic, anti-inflammatory, antihypertensive, and hepatoprotective properties [18, 21]. A study showed that SO, by increasing enzymatic and nonenzymatic antioxidants, has better protective effects against hyperlipidemia and lipid peroxidation in comparison with SFO [20].

It has been shown that sesamin in SO has hepatoprotective effects in animal studies. It can improve steatosis in steatohepatitis by regulating lipid metabolism. To be more specific, this process is done by reducing the mRNA expression of lipogenic enzymes, increasing the expression of enzymes involved in fatty acid oxidation in the liver, and increasing antioxidant capacity [22–24]. A study on rats showed that SO reduces liver enzymes and stated that its protective effects are due to its antioxidant components (phytates, lignans, and vitamin E) that may prevent the formation of free radicals [25]. In addition, it increases the antioxidant activity of liver enzymes [26]. Animal studies have shown that daily supplementation with SO significantly reduces the fat content of the liver and serum and the liver damage caused by oxidative stress [18, 19, 27].

To the best of our knowledge, no human study has been examined so far for the effect of SO on the severity of steatosis and serum liver enzymes in patients with NAFLD. With the high prevalence of NAFLD and by assuming that SO reverses or at least reduces hepatic steatosis, we investigated the effect of replacing regular oil (SFO) with SO on hepatic steatosis and serum liver enzymes in patients with NAFLD on a weight loss diet in a double-blind clinical trial study.

## 2. Materials and Methods

A total of 60 women with NAFLD were recruited in this randomized, double-blind, parallel, and controlled trial in Shahroud, Iran. The current study aimed at assessing the effects of SO on serum liver enzymes, and ultrasonographic indices of hepatic steatosis in women with NAFLD. SPIRIT (Standard Protocol Items: Recommendations for Interventional Trials) was used as a framework for reporting the present protocol [28]. The ethical approval of this trial was obtained from the Ethics Committee of Isfahan University of Medical Sciences on November 9, 2020, with a reference number of IR.MUI.RESEARCH.REC.1399.548, and this trial was registered in the Iranian Registry of Clinical trials (https:/www.irct.ir) with the registration code of IRCT20140208016529N6 on December 12, 2020.

### 2.1. Subjects

A total of 60 women with NAFLD were recruited from patients from the liver and gastrointestinal clinics in Shahroud, Iran. The mean age was 39 years, and the mean BMI was 31.3 kg/m^2^.

Female participants being 20 to 50 years old, having NAFLD by examining ultrasonography, consuming SFO as the routine oil, and having BMI between 25 and 40 were included in the study (inclusion criteria).

The exclusion criteria applied to the potential participants were as follows: having been smokers, alcohol consuming, menopausal, pregnant, breastfeeding; having undergone insulin therapy throughout the study period; having hormone-dependent cysts and allergies, history of breast cancer, sclerosing cholangitis, renal failure, autoimmunity, malignancies, celiac disease, hereditary hemochromatosis (transferrin saturation greater than 45%), Wilson's disease, liver diseases (cirrhosis, alcoholic liver disease, viral hepatitis, hepatitis, primary biliary cirrhosis, biliary obstruction, and liver damage induced by hereditary hemochromatosis drugs); have consumed hepatotoxicity drugs such as tamoxifen and lithium, drugs affecting the levels of liver enzymes ALP, AST, and ALT including valproic acid, 3-hydroxy-3-methylglutaryl coenzyme A reductase inhibitors, acetaminophen, salicylates, phenytoin, benzodiazepines, drugs causing fatty liver such as methotrexate, tamoxifen, and valproate, drugs such as corticosteroids, amiodarone, perhexiline, aspirin, hydralazine, contraceptives, estrogen; have participated in other studies in the last 6 months; having any weight loss diet or special diet in the last three months; and consuming multivitamin mineral and omega-3 supplements three months prior the trial.

The participants who lost to follow up the study for any reason or had improper adherence were excluded from the analysis (dropout criteria).

### 2.2. Study Design

The study was a randomized, double-blind, parallel, controlled trial. 60 individuals were randomly divided into two groups receiving either SO (intervention group) or SFO (control group) using permuted block randomization method with block sizes of four. SFO is widely produced and distributed in Iran and is well known as the main type of oil consumed by Iranians [17]. The types of oils used for the trial and the study objectives were blinded by a person out of this study. The refined, odorless oils were placed in similar, opaque bottles with A and B labels. As a result, participants, facilitators, and researchers became blind to the types of oils being consumed.

At the first visit, all study protocols were explained to the participants, and written consents, demographic information, and their medical histories were obtained. During the run-in, participants used routine oil (SFO) and healthy eating recommendations. After two weeks of run-in, participants were divided into two groups by using random allocation method by a third party who did not know about the study and its objectives: (1) 30 individuals in group A received 30 grams per day of SO and hypocaloric diet and (2) 30 individuals in group B received 30 grams per day of SFO and hypocaloric diet. Then, the intervention was performed for 12 weeks.

### 2.3. Design for the Diet

The estimated energy requirement (EER) for each individual was calculated, using the Mifflin–St Jeor equation [29], and 500 calories were deducted. The energy distributions consisted of 50–55% carbohydrates, 14–18% protein, and 27–32% fat. Food groups and the food-exchanging were explained to the participants. Half of the oil containers were given to the participants at the beginning of the trial for the first 6 weeks of the study and the rest were given after the 6-week period. Calibrated cups were given to individuals to consume the exact amount of 30 grams of oil per day on their cooked foods or salads for 12 weeks. The patients who were determined to be adherent to the trial were identified by the number of containers they returned and also consumed more than 90 percent of oils.

Four clinical visits were performed at the beginning of run-in and at the beginning, middle, and end of the intervention. Participants were asked not to change their recommended diet, physical activity, and medications during the study. Furthermore, they were asked to report any changes and were followed up by telephone each week.

### 2.4. Measurements

#### 2.4.1. Anthropometric Measurements

Body weight was measured using a digital calibrated scale (mode BG 51XXL, Seca, Germany) with light clothes and an accuracy of 0.1 kg. Height was measured in the standing position without shoes while leaning against the wall and shoulders being in normal condition with an accuracy of 0.5 cm using a tape measure mounted on the wall. BMI was calculated based on body weight (kg) divided by height squared (m^2^). WC was measured in the middle area between the lowest rib and the upper iliac bone with a nonstretchable measuring tape at the end of a normal exhalation. To eliminate measurement errors, all measurements are performed by a trained person, three times per visit.

#### 2.4.2. Dietary Intake Assessment

To assess the diet, participants were instructed in a public session by a nutritionist on how to fill out the food records (the type and amount of all consumed foods and beverages). Participants completed the three-day weighted food record forms (two weekdays and one weekend day) at the beginning, middle, and end of the intervention to measure dietary nutrients intake, including energy, macronutrients, and micronutrients intake. A total of nine food records for each individual were analyzed using Nutritionist IV software modified for Iranian foods (version 3.5.2, Axxya Systems, Redmond, Washington, DC, USA).

#### 2.4.3. Physical Activity Assessment

A three‐day self‐report record (two weekdays and one weekend day) was used to assess physical activity at the beginning, middle, and end of the intervention. A total of nine physical activity records were obtained for each individual. The participants were asked to maintain their usual physical activity patterns throughout the study. Physical activity data were converted to metabolic equivalent (MET) hour/day, using the updated compendium of physical activities [30].

#### 2.4.4. Chemical Analysis of SO and SFO

Fatty acid composition of SO and SFO (Kamjed Company, Shahroud, Iran) was evaluated using high-performance gas chromatography at the reference food chemistry laboratory (ViroMed Specialized Laboratories, Tehran, Iran). The percentages of PUFAs, MUFAs, and saturated fatty acids (SFAs) were 52.57%, 35.83%, and 11.6% in the SFO and were 46.57%, 38.25%, and 15.18% in the SO, respectively. The concentrations of *n* − 3 and *n* − 6 fatty acids were 0.16% and 52.41% in SFO and 0.26% and 46.31% in SO, respectively. The fatty acid profiles of both types of oils are shown in Table 1.

#### 2.4.5. Blood Marker Assessment

At the beginning and the end of the intervention, 10 ml of venous blood from the participants' left arm was taken after an overnight fast (12 hours) between 7 AM and 10 AM. The blood samples were taken in the sitting position, at the laboratory of Razavi clinic, Shahroud, Iran. It was centrifuged at 3000 rpm for 5 minutes, and after serum separation, it was kept at −80°C until the analysis. ALP, AST, and ALT hepatic enzymes were measured using the enzymatic colorimetric method.

#### 2.4.6. Ultrasound Imaging of the Liver

At the beginning and the end of the study, a liver ultrasound was performed to assess the severity of the steatosis by a radiologist blinded to the trial details. The liver ultrasound device was LOGIQ S8 (United States). After 8 hours of fasting in the afternoon, the patient was placed in an open position, and the right and left lobes were examined from the upper to the lower surface. Hepatic steatosis was reported semiquantitatively based on liver echo-texture parameters, brightness of the liver, contrast ratio of the liver-to kidney, and blurred vessels, with three degrees, namely, “mild,” “moderate,” and “severe” [31].

### 2.5. Statistical Analyses

Shapiro–Wilk's W test was used to assess the normality of quantitative data distribution. Qualitative and quantitative variables were expressed as frequency report (percentage) and mean ± standard deviation, respectively. For the intragroup analyses, paired sample *t*-test (variables with normal distribution) and Wilcoxon test (variables without normal distribution and qualitative variables) were used for comparing the mean of variables. For the intergroup analyses, independent sample *t*-test and Mann–Whitney test were used to examine the mean of quantitative variables with and without normal distribution, respectively. However, chi-square test was used for qualitative variables. Analysis of covariance (ANCOVA and nonparametric ANCOVA) was used to compare the changes of quantitative variables between two groups in the presence of confounders. However, binary logistic regression was used for steatosis improvement (qualitative variable). The potential confounders of baseline BMI, physical activity changes, energy intake changes, and baseline values of the variables were included as covariates in the univariate-adjusted model. *P* value < 0.05 was considered statistically significant. SPSS software (version 25, SPSS Inc., Chicago, IL, USA) was used for data analyses.

The sample size was calculated for parallel clinical trial studies containing an intervention and a control groups. According the standard formula for clinical trials [32], a sample size of 30 patients in each group was determined based on the changes in mean of fatty liver grade as the primary outcome (0.46) and standard deviations (SD1 = 0.45 and SD2 = 0.46) as reported by Rezaei et al. [33] with the assumptions of the type I error (*α*) of 0.01, type II error (*β*) of 0.2 (power = 80%), and around 20% dropouts.

## 3. Results

**Figure 1:**
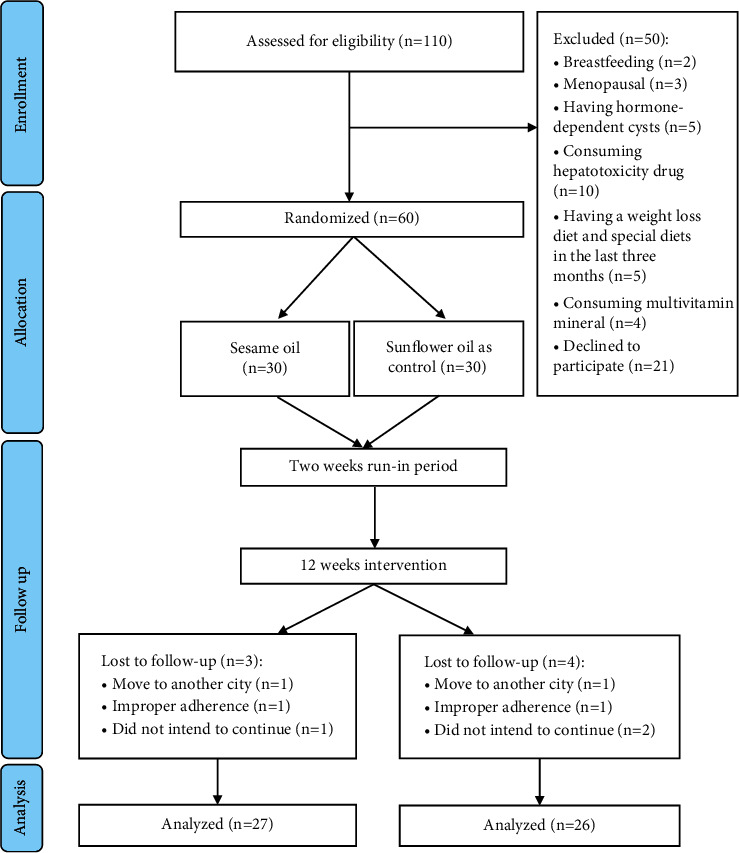
Flowchart of patient recruitment for the clinical trial.

Of the total 110 patients with NAFLD assessed for eligibility, 50 patients were excluded based on the exclusion criteria, and 60 participants enrolled, gave their informed written consents, and participated in the trial. They were randomly divided into either the SO group as the intervention group (*n* = 30) or the SFO group as the control group (*n* = 30). Three subjects from the intervention group and four subjects from the control group dropped out during the intervention: adhered improperly (*n* = 2), moved to another city (*n* = 2), and did not intend to continue (*n* = 3). In total, 53 subjects completed this trial and were included in the analysis (Figure 1). No side effects were observed from oil consumption during the intervention period.

There were no significant differences in physical activity and dietary intake (Table 2), age, education level, body weight, BMI, WC, serum AST, ALP, and ALT levels, and fatty liver grade (Table 3) between the two groups at the beginning.

The averages of mid‐ and postintervention values for dietary intake and physical activity of each group are presented in Table 2. There was a significant decrease in levels of total energy, carbohydrates, proteins, fat, PUFAs, MUFAs, and SFAs in each group after the intervention. At the end of the intervention, the level of MUFAs in the SO group was significantly higher than that in the control group (*P* < 0.05), and the level of PUFAs in the control group was significantly higher than that in the SO group (*P* < 0.001). Physical activity remained unchanged throughout the study period in both groups. After the intervention, no significant differences between the two groups were observed for total energy and macronutrient and micronutrient intake except for vitamin E (*P* < 0.05). Vitamin E increased significantly in the SO group compared to the SFO group.

**Figure 2:**
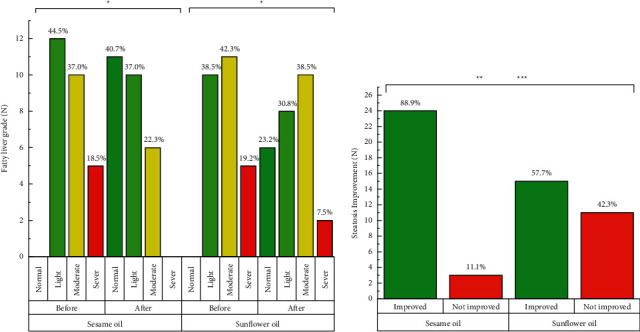
Changes in hepatic steatosis after 12 weeks of intervention. ^*∗*^ denotes significant *P* value for intragroup analysis. ^*∗∗*^ denotes significant *P* value for between-group comparison for crude model. ^*∗∗∗*^ denotes significant *P* value for between-group comparison for adjusted model (baseline BMI, physical activity changes, energy intake changes, and baseline values of the variable).

By giving a weight loss diet at the beginning of the study, a significant body weight reduction was observed in the SO group (−4.59 kg) and the control group (−3.97 kg) after the intervention (*P* < 0.001) (Table 4). At the end of the study, fatty liver grade significantly decreased in both groups (Figure 2). The reduction in the SO group was much more than that in the control group (89.9% vs. 57.7%), which led to a significant difference between the two groups in both unadjusted and adjusted models (*P* < 0.05).

**Figure 3:**
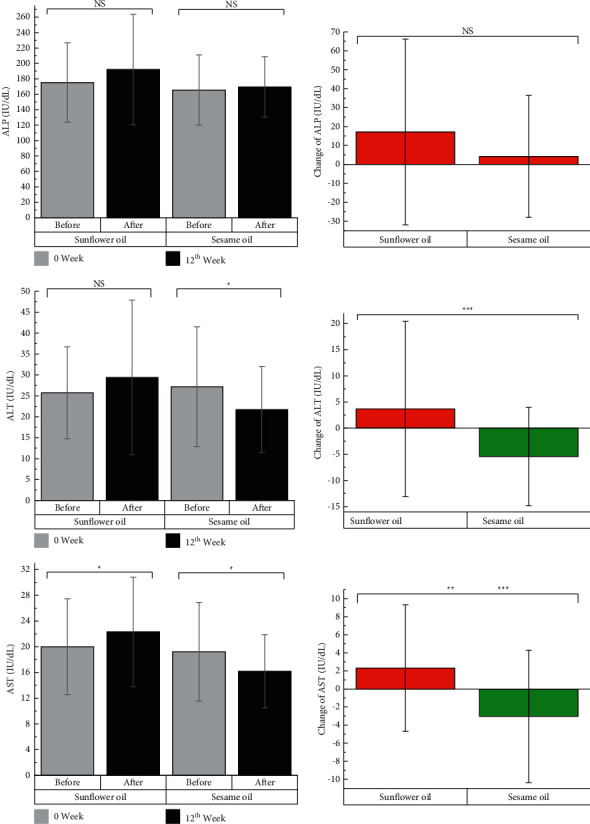
Changes in the levels of liver enzymes after 12 weeks of intervention. Changes imply for after minus before. ^*∗*^ denotes significant *P* value for intragroup analysis. ^*∗∗*^ denotes significant *P* value for between-group comparison for crude model. ^*∗∗∗*^ denotes significant *P* value for between-group comparison for adjusted model (baseline BMI, physical activity changes, energy intake changes, and baseline values of the variable). NS: not significant; ALT: alanine aminotransferase; AST: aspartate aminotransferase; ALP: alkaline phosphatase.

The analyses of liver function values such as ALP, ALT, and AST are shown in Figure 3. The intragroup assessment of liver enzymes revealed an improvement in serum ALT and AST levels in the SO group (*P* < 0.05). Furthermore, at the end of the study, significant decreases were observed in the serum ALT and AST levels in the SO group compared to the control group (<0.05). No significant change in the serum ALP level was observed in the intragroup and intergroup analyses at the end of the study.

## 4. Discussion

To the best of our knowledge, the present study is the first randomized controlled trial examining the effect of SO in the context of a weight loss program with normal fat content (27%–32%) on serum liver enzymes and hepatic steatosis in patients with NAFLD. In addition, the present study showed that the consumption of 30 g of SO for 12 weeks significantly reduced fatty liver grade. Similarly, this reduction was significant in the control group, but the improvement was significantly much higher in the SO group than in the control group. In addition, consumption of SO significantly reduced ALT and AST, which were significantly higher compared to the control group.

The assessment of dietary intake after the intervention showed that vitamin E intake was significantly increased in the SO group compared to the control group. Also, MUFAs in the SO group were significantly higher than those in the control group, and PUFAs in the control group were significantly higher than those in the SO group.

### 4.1. Fatty Liver

Our study showed hepatic steatosis improvement in the SO group was more than that in the control group. Weight loss, another factor in the first line of treatment in patients with NAFLD, can effectively improve steatosis [34]. Body weight significantly reduced in both groups at the end of our study, and some studies have observed this effect [35, 36].

There are no human studies that examined the effect of SO on hepatic steatosis in NAFLD patients. Several animal studies investigated the effect of SO on hepatic steatosis and showed promising results [3, 19, 20, 22, 37]. Yang et al. reported that the SO consumption for 8 weeks ameliorated hepatic lipid accumulation in mice with NAFLD [22]. Periasamy et al.'s study showed supplementation of 4 ml/day of SO for 35 days protected mice against fibrosing steatohepatitis by inhibiting matrix metalloproteinases-2, 9 activities and upregulating PPAR-*γ* expression [20].

The type and amount of dietary fats play an important role in fat accumulation in the liver and are responsible for 15% of the liver fat content [38]. SO contains a high proportion of oleic acid [19]. High-speed oxidation of MUFAs compared to SFAs can have beneficial effects on the hepatic fat content [39]. MUFAs are deposited more in adipose tissue than in the liver, and a diet rich in MUFAs may help to prevent fat deposition in the liver [38].

The antioxidative capacity of SO can scavenge the free radicals and reduce the damage to liver cells and protect liver mitochondria [40]. Sankar et al. reported that SO is a good source of antioxidants such as phenols, sesamin, and vitamin E that has better protective effects against hyperlipidemia and lipid peroxidation by increasing enzymatic and nonenzymatic antioxidants compared to SFO [41]. Our study showed that SO consumption is better than SFO consumption in improving hepatic steatosis.

The phytochemicals such as sesamin, sesamolin, and sesamol in SO can improve steatosis in the liver by regulating lipid metabolism and increasing antioxidant capacity [22]. Some studies have shown that they reduce the mRNA expression of lipogenic enzymes in the liver such as fatty acid synthase, pyruvate kinase, glucose 6-phosphate dehydrogenase, and ATP citrate lyase and increase the mRNA expression of enzymes involved in fatty acid oxidation such as carnitine palmitoyltransferase, acyl-CoA dehydrogenases, acyl-CoA oxidase, 3-hydroxyacyl-CoA dehydrogenase, enoyl-CoA hydratase, and 3-ketoacyl-CoA thiolase. Also, SO can reduce the action of phosphatidate phosphohydrolase enzyme, thereby reducing the synthesis of triglycerides and the risk of fatty liver in hypercholesterolemic diets [23, 24]. Sesamin lignan in SO inhibits the delta-5 desaturases enzyme that catalyzes the desaturation of gamma-linolenic acid (20 : 3*n* : 6) to arachidonic acid (20 : 4*n* : 6) [42].

### 4.2. Liver Enzymes

In our study, SO mitigated ALT and AST levels in participants with NAFLD. There are only two human studies that examined the effect of SO on liver enzymes. Our findings are in line with findings in a parallel study, consuming 30 ml/day SO for 90 days on patients with type 2 diabetes showing a significant decrease in AST and ALT levels [26]. However, in a cross‐over trial examining the effects of SO for 12 weeks on liver enzymes on patients with type 2 diabetes, no significant changes were observed in ALP, AST, and ALT levels [17]. The difference between the results of our study and this study may be due to the differences in participants' disease, dosage of SO, and study design.

Our findings are in line with findings in some animal studies. Consumption of a diet containing 12% SO for 60 days increased ALP levels and decreased AST and ALT levels in rats [43]. Also, intake of SO (150 mg/kg per day) for 30 days decreased AST and ALT levels in male rats [25]. Similar results were observed in other animal studies [27, 44].

There are several reasons for beneficial effects of SO on reducing hepatic damage and the levels of AST and ALT enzymes: (1) the protective effects of SO are due to its antioxidant compounds such as lignans, vitamins E, phytate, pinoresinol, and lecithin, which may prevent free radical formation and scavenge them [25, 45]; (2) SO increases secretion of bile salt, AST, and ALT in the liver; (3) flavonoids and antioxidant in SO transform vitamins B6 to pyridoxal 5-phosphate, which acts as a coenzyme for aminotransferases; and (4) SO lignans increase the hepatic mitochondria and rate of peroxisomal fatty oxidation and active *α*-oxidase cycle (*α*-oxidation is important in the catabolism of branched-chain fatty acids) and protect liver function [25].

Changes in liver enzyme levels may have multifactorial origins [46]. Nutritional genomics has a key role in gene-diet interaction in the occurrence and progression of NAFLD [47]. For example, it is suggested that FTO gene levels in the liver are involved in lipid deposition that may lead to NAFLD [48]. Its expression is related to the type and amount of macronutrients [49]. Interestingly, the gene-diet interaction can affect the success of lifestyle interventions in the prevention and treatment of NAFLD.

### 4.3. Strengths and Limitations

Our study has several strengths: (1) the methodology and design of previous studies were not rigorous due to the lack of allocation concealment, blinding of participants and personnel, physical activity, and dietary intake assessments, and we did all of them to minimize the potential risk of biases; (2) all participants were female and aged 20–50 years who were almost homogeneous in physiological and hormonal conditions; and (3) we used healthy vegetable oils and weight loss diets in both groups and hepatic steatosis improvement in the SO group was more than that in the control group.

Our study has several limitations that should be taken into account when interpreting the results: (1) We did not examine the fatty acid content of red blood cells (RBC) and serum levels of vitamin E. Therefore, future research is needed to use more objective methods of assessing the type of fatty acids (MUFAs and PUFAs) in RBC and serum levels of vitamin E to confirm adherence [50, 51]. (2) We did not include oils with high SFAs such as hydrogenated oils and palm oil found in the western diet. It does not seem ethical to use unhealthy oils for a long time (12 weeks) in clinical trials. (3) Ultrasonography has been accepted as the first and best diagnostic method for NAFLD in the investigations [9], and we blinded radiologist to the group allocation of the patients to reduce unwanted errors in grading fatty liver, but ultrasound is not very accurate in the detection of mild cases of fatty liver (degree of fat infiltration less than 30%). Its results depend on expertise and skills of the operator and sensitivity of ultrasonography [10].

## 5. Conclusions

The results of this trial suggest that the desired effects of weight loss were reinforced by the consumption of a low-calorie diet enriched with SO through improving fatty liver severity and serum ALT and AST levels. Also, the low-calorie diets may lead to favorable effects for NAFLD patients by mitigating obesity and fatty liver grade. Future studies are needed to assess the effects of SO on fatty liver undergoing an isocaloric diet and it is recommended to use fibroscan instead of sonography for detecting and tracking hepatic steatosis due to its high sensitivity and accuracy.

## Figures and Tables

**Table 1:** Fatty acid composition of sunflower and sesame oils.

Fatty acids	Sesame oil	Sunflower oil
C16 : 0	9.58	6.33
C18 : 0	4.92	4.25
C20 : 0	0.5	0.31
C22 : 0	0.18	0.71
C18 : 1	38.25	35.83
C18 : 2	46.31	52.41
C18 : 3	0.26	0.16
∑SFA	15.18	11.6
∑MUFA	38.25	35.83
∑PUFA	46.57	52.57

All values are percentage of total fatty acids. SFAs: saturated fatty acids; MUFAs: monounsaturated fatty acids; PUFAs: polyunsaturated fatty acids.

**Table 2:** Physical activity and dietary intake of study participants at baseline and after intervention.

		Sesame oil (*n* = 27)	Sunflower oil (*n* = 26)	*P* value^*∗∗*^
Variable	Status	Mean ± std. deviation	Mean ± std. deviation	
Energy (kcal/day)	Before	2166.82 ± 576.94	2258.53 ± 529.81	0.423
	After	1590.83 ± 261.87	1702.95 ± 322.94	0.294
	*P* value^*∗*^	<0.001	<0.001	

Physical activity (MET hour/day)	Before	33.55 ± 3.65	33.22 ± 3.54	0.734
	After	33.58 ± 3.57	33.42 ± 2.90	0.858
	*P* value^*∗*^	0.883	0.562	

Protein (g/day)	Before	78.30 ± 17.31	80.69 ± 25.09	0.687
	After	70.48 ± 13.02	69.63 ± 11.43	0.803
	*P* value^*∗*^	0.035	0.028	

Carbohydrates (g/day)	Before	290.75 ± 115.57	307.33 ± 95.64	0.311
	After	207.01 ± 44.40	226.85 ± 50.41	0.160
	*P* value^*∗*^	0.001	<0.001	

Fat (g/day)	Before	81.68 ± 15.97	88.80 ± 16.20	0.070
	After	57.47 ± 7.37	61.55 ± 12.59	0.393
	*P* value^*∗*^	<0.001	<0.001	

SFAs (g/day)	Before	24.36 ± 8.06	26.10 ± 7.28	0.286
	After	15.25 ± 3.39	15.38 ± 4.45	0.709
	*P* value^*∗*^	<0.001	<0.001	

MUFAs (g/day)	Before	24.83 ± 4.57	26.19 ± 4.46	0.122
	After	19.65 ± 2.33	18.85 ± 3.49	0.041
	*P* value^*∗*^	<0.001	<0.001	

PUFAs (g/day)	Before	27.07 ± 5.29	28.99 ± 6.55	0.247
	After	19.81 ± 2.57	23.84 ± 5.87	<0.001
	*P* value^*∗*^	<0.001	0.001	

Fiber (g/day)	Before	7.74 ± 3.23	8.10 ± 2.93	0.569
	After	6.63 ± 1.98	6.87 ± 1.45	0.615
	*P* value^*∗*^	0.077	0.066	

Beta-carotene (*μ*g/d)	Before	987.77 ± 2199.16	1012.47 ± 1208.32	0.233
	After	634.86 ± 857.30	934.50 ± 987.35	0.075
	*P* value^*∗*^	0.701	0.657	

Vitamin E (mg/day)	Before	2.63 ± 1.60	3.64 ± 2.25	0.089
	After	11.34 ± 1.14	2.65 ± 1.06	<0.001
	*P* value^*∗*^	<0.001	0.038	

Vitamin C (mg/day)	Before	85.96 ± 45.06	112.10 ± 113.81	0.817
	After	68.68 ± 35.34	105.34 ± 74.29	0.311
	*P* value^*∗*^	0.097	0.909	

Selenium (mg/day)	Before	0.003 ± 0.008	0.003 ± 0.007	0.765
	After	0.002 ± 0.004	0.001 ± 0.003	0.484
	*P* value^*∗*^	0.173	0.645	

Zinc (mg/day)	Before	10.25 ± 3.05	10.48 ± 3.49	0.986
	After	8.69 ± 1.85	8.44 ± 1.86	0.466
	*P* value^*∗*^	0.019	0.034	

Intragroup analysis: *P* value^*∗*^ reported based on paired sample *t*-test and Wilcoxon test. Between-group comparison: *P* value^*∗∗*^ reported based on independent sample *t*-test and Mann–Whitney test. SFAs: saturated fatty acids; PUFAs: polyunsaturated fatty acids; MUFAs: monounsaturated fatty acids; MET: metabolic equivalent.

**Table 3:** Baseline characteristics of the participants.

Quantitative variable		Sesame oil (*n* = 27)	Sunflower oil (*n* = 26)	*P* value^*∗∗*^
		Mean ± std. deviation	Mean ± std. deviation	
Age (years)		38.89 ± 6.91	39.35 ± 5.89	NS
Height (cm)		160.96 ± 4.37	161.02 ± 6.19	NS
Body weight (kg)		79.94 ± 9.57	82.91 ± 13.77	NS
BMI (kg/m^2^)		30.85 ± 3.45	31.86 ± 4.13	NS
WC (cm)		106.39 ± 9.71	108.19 ± 9.93	NS
AST (IU/dL)		19.22 ± 7.67	20.00 ± 7.45	NS
ALP (IU/dL)		165.44 ± 45.45	175.08 ± 51.53	NS
ALT (IU/dL)		27.19 ± 14.32	25.73 ± 11.00	NS
Qualitative variable		*N* (percent)	*N* (percent)	*P* value^*∗∗*^
Education	High school	5 (18.50)	10 (38.50)	NS
	Diploma	11 (40.75)	9 (34.60)	
	Bachelor	11 (40.75)	7 (26.90)	
Fatty liver grade	Normal	0 (0)	0 (0)	NS
	Light	12 (44.50)	10 (38.50)	
	Moderate	10 (37.00)	11 (42.30)	
	Severe	5 (18.50)	5 (19.20)	

Between-group comparison: *P* values^*∗∗*^ were reported based on independent sample *t*-test and Mann–Whitney test for quantitative variables and chi-square test for qualitative variables. NS: not significant; ALT: alanine aminotransferase; AST: aspartate aminotransferase; ALP: alkaline phosphatase; BMI: body mass index; WC: waist circumference.

**Table 4:** Changes in anthropometric variables after 12 weeks of intervention.

		Sesame oil (*n* = 27)	Sunflower oil (*n* = 26)	*P* value^*∗∗*^	*P* value^*∗∗∗*^
Quantitative variable	Status	Mean ± std. deviation	Mean ± std. deviation		
Body weight (kg)	After	75.35 ± 9.70	78.94 ± 13.63	0.223	0.154
	Change	−4.59 ± 2.26	−3.97 ± 1.79		
	*P* value^*∗*^	<0.001	<0.001		

BMI (kg/m^2^)	After	29.07 ± 3.44	30.32 ± 4.09	0.255	0.165
	Change	−1.78 ± 0.90	−1.53 ± 0.69		
	*P* value^*∗*^	<0.001	<0.001		

WC (cm)	After	100.48 ± 9.31	103.62 ± 9.73	0.135	0.059
	Change	−5.91 ± 3.77	−4.57 ± 2.26		
	*P* value^*∗*^	<0.001	<0.001		

Changes imply for after minus before. Intragroup analysis: *P* values^*∗*^ were reported based on paired sample *t*-test. Between-group comparison for crude model: *P* values^*∗∗*^ were reported based on Mann–Whitney test. Between-group comparison for adjusted model (baseline BMI, physical activity changes, energy intake changes, and baseline values of the variable): *P* values^*∗∗∗*^ were reported based on nonparametric ANCOVA. BMI: body mass index; WC: waist circumference.

## Data Availability

The data used to support the findings of this study are available from the corresponding author upon request.
